# Editorial: Mitochondria-Targeted Nanocarriers for Enhanced Efficacy of Cancer Therapy

**DOI:** 10.3389/fbioe.2022.905999

**Published:** 2022-05-31

**Authors:** Yan Gao, Bing Xia

**Affiliations:** Key Laboratory of Forest Genetics and Biotechnology (Ministry of Education of China), College of Science, Nanjing Forestry University, Nanjing, China

**Keywords:** mitochondria targeting, nanomedicine, enhanced efficacy, cancer therapy, chemotherapy, phototherapy

The main focus of this Research Topic is mitochondria-targeted nanocarriers or nanomedicine *via* the conjugation of mitochondria-directing entities, for enhanced efficacy of cancer therapy, and even the further translation into clinical practice. Mitochondria are subcellular compartments playing an important role by supplying ATP and participating in regulation of signaling pathways, including the regulation of cell cycles such as differentiation and necrosis (Roger et al., 2017). The abnormalities of mitochondria are considered to result in a variety of diseases (Murphy and Smith, 2000). The dysfunctions or failures of mitochondria are also closely related to cancer occurrence, metastasis, or recurrence (Porporato et al., 2014). Therefore, there is a growing interest in investigating specific mitochondria-targeted therapeutic approaches to improve the efficacy of cancer therapy.

Nanoparticles are versatile and powerful tools for precise cancer therapy, with a passive or active tumor-targeted effect. However, the major drawback of nanomedicine lies in insufficient anticancer therapeutic efficacy. Thus, in order to overcome this limitation, many researchers have gone beyond delivering drugs and therapeutic agents into tumor sites and cancer cells, but rather into subcellular compartments. Considering that the mitochondrion is one of the vital targets of subcellular organelles, multifunctional nanoplatforms are being developed for cancer therapy with the modification of various mitochondria-targeted ligands including lipophilic cations and peptides, etc. On this basis, mitochondria-targeted precise cancer therapy can be enabled by these innovatively designed nanoplatforms integrated with more diagnostic and therapeutic modalities.

In the review “*Mitochondria-Targeted Nanomedicine for Enhanced Efficacy of Cancer Therapy*”, Gao et al. elaborated the mechanism by which nanomedicine targeting of mitochondria can enhance the efficacy of cancer therapy and introduced several types of mitochondria-targeting ligands. Cancer therapeutic modalities based on mitochondria-targeted nanomedicines were specified including chemotherapy, photothermal therapy (PTT), photodynamic therapy (PDT), chemodynamic therapy (CDT), sonodynamic therapy (SDT), radiodynamic therapy (RDT), and combined immunotherapy. Moreover, some ongoing theories such as mitochondria may influence the macrophage polarization by regulating intracellular ROS levels to participate in immunotherapy were discussed. And mitochondria-targeting nanosystems were also categorized into lipophilic cation- and peptide-based types, and how mitochondria-targeted nanocarriers promoted highly efficient cancer treatment of PDT, chemotherapy, combined immunotherapy, and SDT was further described (Zeng et al.). In the review by Hu et al., designing multifunctional mitochondria-targeting nanosystems for enhanced anticancer efficacy was highlighted. These multifunctional mitochondria-targeting nano drug delivery systems (DDS) included DQAsomes, liposomes, inorganic nanoparticles, polymeric nanomicelles, and DNA nanostructures. The authors also presented several future prospects, such as the development of multistage and biocompatible nanosystems with multi-targeting of the tumor cell membrane and mitochondria.

Unlike previous more broad reviews, some reviews focused on specific nanoparticles and their applications for mitochondria-targeted cancer therapy. For example, polymer-based nanoparticles for cancer therapy stand out with their great biocompatibility, readily design and synthesis, and flexible ligand conjugation. “*Polymeric Nanoparticles for Mitochondria Targeting-Mediated Robust Cancer Therapy*” by Sun et al. discussed the smart design of polymeric nanoparticles by modifying mitochondria-targeting ligands like lipophilic cations, peptides, and aptamers to deliver various therapeutic agents to mitochondria. The drug-loaded polymeric nanoparticles exhibited a rapid and precise localization in mitochondria, leading to significantly higher cancer therapy effects. In addition, self-assembled peptide materials also excel due to their biosafety, biocompatibility, biomarker targeting, and stimuli response, rendering them competitive candidates for delivering drugs and therapies to mitochondria. The study “*Mitochondria-Targeted Self-Assembly of Peptide-Based Nanomaterials*” was undertaken by Luo et al. It highlighted the recent progress of mitochondria-targeted peptide nanomaterials, constructing self-assembling peptides that target and assemble in mitochondria, including the introduction of mitochondria-targeted ligands, a stimuli-responsive mechanism to trigger *in situ* self-assembly systems in mitochondria, and their applications in cancer treatments.

Mitochondria-targeted cancer therapy is not only limited to the enrichment of nanomedicines or nanocarriers near the mitochondria area through the mitochondria-targeted moieties or ligands, but also includes disturbing the functionality of mitochondria, such as suppressing energy metabolism by disrupting the oxidation respiratory chain. In this scope, an RGD-modified self-assembling peptide composed of an anticancer drug including pemetrexed (PEM) and phenylalanine-phenylalanine (PEM-FFRGD) was prepared to deliver PEM to tumors by Liu et al. The peptide-based nanoparticle showed stronger cytotoxic activity than PEM alone on lung cancer cell lines, with superior solubility and biocompatibility compared with those of PEM. This elevated cancer cell inhibition effect was attributed to the suppression of the energy metabolism of PEM-FFRGD, which occurred mainly in mitochondria. Damage to mitochondrial function or the electron transport chain will directly lead to an increase in ROS levels in cells, which finally lead to tumor necrosis. The enhanced antitumor efficacy of PEM-FFRGD was also validated *in vivo* studies.

Finally, in the scope of mitochondria-targeted cancer therapy, the mitochondria membrane is an essential and key point of focus. “*Mitochondrial membrane remodeling*” is a review conducted by Yang et al. They presented the reorganization of the mitochondria outer membrane during fusion and fission at the molecular level, the factors influencing the mitochondrial inner membrane curvature, and the mitochondria outer and inner membrane functional association with human diseases.

Taken together, these articles highlight that nanocarriers or nanomedicines targeting mitochondria are a promising approach for enhancing the efficacy of anticancer therapy.

## References

[B1] Murphy M. P. Smith R. A. J. (2000). Drug Delivery to Mitochondria: the Key to Mitochondrial Medicine. Adv. Drug Deliv. Rev. 41, 235–250. 10.1016/S0169-409X(99)00069-1 10699318

[B2] Porporato P. E. Payen V. L. Pérez-Escuredo J. De Saedeleer C. J. Danhier P. Copetti T. (2014). A Mitochondrial Switch Promotes Tumor Metastasis. Cell Rep. 8, 754–766. 10.1016/j.celrep.2014.06.043 25066121

[B3] Roger A. J. Muñoz-Gómez S. A. Kamikawa R. (2017). The Origin and Diversification of Mitochondria. Curr. Biol. 27, R1177–R1192. 10.1016/j.cub.2017.09.015 29112874

